# Progresses in Synthesis and Application of SiC Films: From CVD to ALD and from MEMS to NEMS

**DOI:** 10.3390/mi11090799

**Published:** 2020-08-24

**Authors:** Mariana Fraga, Rodrigo Pessoa

**Affiliations:** 1 Instituto de Ciência e Tecnologia (ICT), Universidade Federal de São Paulo (Unifesp), São José dos Campos SP 12231-280, Brazil; 2 Laboratório de Plasmas e Processos (LPP), Instituto Tecnológico de Aeronáutica (ITA), São José dos Campos SP 12228-900, Brazil

**Keywords:** silicon carbide, chemical vapor deposition, atomic layer deposition, microelectromechanical systems, nanoelectromechanical systems

## Abstract

A search of the recent literature reveals that there is a continuous growth of scientific publications on the development of chemical vapor deposition (CVD) processes for silicon carbide (SiC) films and their promising applications in micro- and nanoelectromechanical systems (MEMS/NEMS) devices. In recent years, considerable effort has been devoted to deposit high-quality SiC films on large areas enabling the low-cost fabrication methods of MEMS/NEMS sensors. The relatively high temperatures involved in CVD SiC growth are a drawback and studies have been made to develop low-temperature CVD processes. In this respect, atomic layer deposition (ALD), a modified CVD process promising for nanotechnology fabrication techniques, has attracted attention due to the deposition of thin films at low temperatures and additional benefits, such as excellent uniformity, conformability, good reproducibility, large area, and batch capability. This review article focuses on the recent advances in the strategies for the CVD of SiC films, with a special emphasis on low-temperature processes, as well as ALD. In addition, we summarize the applications of CVD SiC films in MEMS/NEMS devices and prospects for advancement of the CVD SiC technology.

## 1. Introduction

An exciting and expanding area of research in materials science involves the development of wide-bandgap (WBG) semiconductor materials that are used to fabricate micro/nanoelectromechanical systems (MEMS/NEMS) for harsh environment sensing applications [1,2]. A goal is to establish synthesis methods that allow controlling the properties of these materials, such as chemistry, physics, and morphology, and consequently, the performance of the sensors and devices constructed with them. The WBG semiconductor materials of current interest for MEMS/NEMS are based on carbon, such as diamond and silicon carbide (SiC), and on the III-nitrides group, such as gallium nitride (GaN) and aluminum nitride (AlN) [3]. SiC has been standing out because of its superior physical and mechanical properties, including wide (tunable) bandgap, low density, high strength and thermal conductivity, structural stability at high environmental temperature, low thermal expansion, high refractive index, high resistance to shocks and chemical inertness, associated to compatibility of its process technology with the silicon MEMS technology, as well as by its well-established synthesis using chemical vapor deposition (CVD) processes [4].

The development of process strategies for CVD synthesis of SiC has been driven by the potential to produce thin and thick films of high-purity and low-density defects and high-quality structured surfaces. In this context, the versatility, quality, and cost-effectiveness of CVD SiC technology for MEMS devices are frequently reported in literature, especially for the fabrication of SiC and SiC/Si suspended membranes and cantilever beams for use in applications, such as pressure sensors, electromechanical resonators, accelerometers, and gyroscopes [5].

Among the various polytypes of SiC, mono- and polycrystalline 3C-SiC have been the most investigated for MEMS applications, being used both as a structural material and as a sacrificial layer to fabricate devices [6]. In the first reports on SiC MEMS, the atmospheric pressure chemical vapor deposition (APCVD) was the technique used for the growth of 3C-SiC epitaxial films. Because of its design and types of precursors, this technique required high temperatures (above 1050 °C), which limited the SiC film growth on a wide range of materials, besides not being suitable for monolithic integration with integrated circuits (ICs) [7]. Driven by these limitations, several efforts have been made to develop methods to synthesize SiC films at lower temperatures than those used in APCVD, such as low-pressure chemical vapor deposition (LPCVD) and plasma-enhanced chemical vapor deposition (PECVD). These methods have enabled the growth of SiC films with uniform thickness, high purity, conformal step coverage, and low cost [8]. Because of advances in PECVD SiC films, studies on the synthesis, properties, and MEMS application of amorphous SiC (a-SiC) have been carried out [9].

Nowadays, CVD SiC films are grown on a variety of substrates, such as silicon, silicon dioxide, SOI (silicon-on-insulator), sapphire, and diamond. In addition to progress in low-temperature CVD processes, atomic layer deposition (ALD) of SiC has been reported more recently [10]. Although substantial challenges permeate the transition from CVD to ALD processes in order to allow the deposition of conforming films on high-aspect-ratio (HAR) nanostructures, great advances have been demonstrated in recent years for devices in thin films of Si-based materials [11]. Currently, there are no known reports of ALD processes for SiC at temperatures compatible with semiconductor device fabrication. However, some research has been carried out in order to adapt the processes of growth of SiO_2_ or SiN_x_ films to SiC films [11,12]. This review will present in detail all these works.

The ALD technique is similar to CVD but distinguished by the fact that it exposes the substrate to the precursors separately; that is, there is no interaction between the precursors in the reactor volume, only on the substrate surface/wall of the reactor. Thus, the film growth mechanism is self-limiting, i.e., the reactions between the precursor and the substrate surface will automatically cease when all reactive groups on the surface (OH radicals) are filled, thus requiring another precursor for the reaction to continue [13]. Therefore, this technique is categorized by alternately exposing the sample surface to different types of precursor gases and at each exposure approximately one atomic monolayer is deposited, allowing the formation of highly conformal and ultrathin films. In general, two different sources of vapors or precursor gases are used in ALD-based processes. These are sequentially inserted into the reaction chamber where, between each exposure, this is purged with a nonreactive gas or evacuated. This prevents unwanted reactions in the gas phase from being prevented from occurring and in parallel removes byproducts from the ALD chamber. The precursor chemistry is adjusted to guarantee saturation of the surface with the respective precursor molecules subsequently to each half cycle. For example, for the deposition of a monolayer of silicon oxide (SiO_2_) material, a flow of Si precursor is first inserted; then purging is done; after purging, the oxidizing precursor is inserted, and finally it is purged again. This sequence of steps is called the ALD deposition cycle [14]. ALD processes are commonly divided into two types, purely thermal or plasma-assisted (PEALD). In the case of PEALD, radicals and ions generated during the plasma half-cycle allow the growth reactions of the film to occur at a lower process temperature and short exposure time of the metal precursor [15].

As the critical dimensions of the SiC-based devices continue to decrease [16], the CVD and PECVD processes became hard to integrate due to the possibility of pinch-off in the HAR structures [11]. Thus, turning to ALD can be a potential alternative for SiC-based NEMS devices.

In this review article, the latest progress and trends in CVD synthesis methods of SiC films for fabricating MEMS/NEMS devices are highlighted. First, we present the evolution of the CVD processes used for SiC film growth up to the promising ALD. In the subsequent section, we summarize the MEMS/NEMS devices and sensors based on CVD SiC films.

## 2. Chemical Vapor Synthesis of SiC Films: From CVD to ALD

CVD is a process for making solids from gases. CVD dates back to the 1950s, when it was first introduced by Union Carbide for the synthetic production of diamonds [17]. Although interest in CVD synthesis of diamond has declined in recent years, interest in CVD techniques has increased for the production of other materials, such as silicon-based materials deposition for use in electronics [18]. In CVD growth, molecular gases are inserted in the reaction chamber where, subsequently, they are dissociated and/or reacted onto the substrate surface in order to grow a film. A wide variety of systems are employed in this film growth technique, which has the following requirements: to be economically viable; to produce high-quality films, i.e., with excellent uniformity, purity of the synthesized material, step coverage and adhesion; high throughput, etc. CVD’s science and technology include special knowledge in numerous fields of science and engineering, namely precursor and growing material chemistry, thermodynamics, technological plasmas, fluid dynamics, radio frequency (RF) power supply technology and characteristics, vacuum technology, and mechanical design [19].

In the CVD process, the reactants originated from precursors undergo surface migration to the reactor/substrate surface sites, where the growth of the film occurs. After the reaction, the byproducts are transported to the outlet of the CVD chamber. In general, CVD processes that occur at low pressure (LPCVD) are carried out in a limited mode of reaction with the surface; that is, this reaction is dependent on substrate temperature following the expression of Arrhenius exp(−*E*_a_/*kT*), where *E*_a_ is the activation energy, *k* is the Boltzmann constant, and *T* the temperature [19]. To guarantee the uniformity of the film being grown, it is essential to maintain a uniform temperature over the entire surface of the substrate. Therefore, the influx rate of reactant species to the substrate surface is not so important, as the design of the reactor can generally ensure enough gas feed in all important points of the chamber. For APCVD processes, the reverse is true because as the mass transport is limited, the temperature control is not critical [8]. All reactant species that reach the substrate surface will participate in the film formation reaction [20].

**Figure 1:**
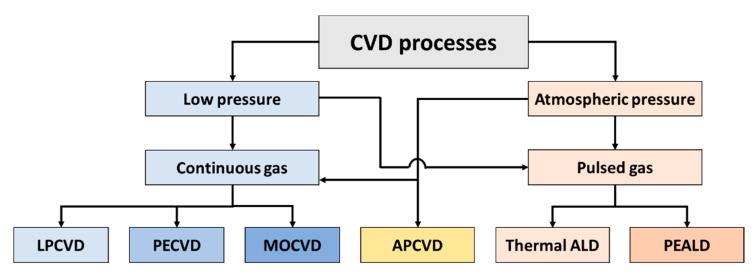
Main types of chemical vapor deposition (CVD) reactors and processes.

There are several models of CVD reactors and processes, as summarized in Figure 1. In practice, the reactors can be of two types: cold or hot wall, meaning that the reactor walls have lower temperatures or the same order of magnitude as the substrate. They can operate in the atmospheric pressure (APCVD) or sub atmospheric pressure, which is generally in the range of 1 Torr to 10 mTorr. In addition, for LPCVD systems, the growth reaction energy is purely thermal or enhanced with the use of a plasma source. The last type of CVD reactor is termed as plasma-enhanced chemical vapor deposition (PECVD). Another type of CVD reactor operated at low pressure is the metal–organic CVD (MOCVD) that is done by flowing a reactive metal–organic precursor vapors over the heated substrates. Regarding atmospheric and low-pressure CVD processes, it is important to note that they operate at continuous gas feed; that is, as an “analogical type mode”, a fact that creates a series of difficulties in controlling film thickness and conformity on different types of substrates, thus limiting the use of these techniques in applications that require films with ultra-thin thicknesses (below 100 nm). In this sense, the idea of carrying out self-limited reactions of precursors at the surface level has been an ingenious and effective solution, and for that, it is necessary to insert the precursors in the reactor separately so as not to promote the reaction between them in the reactor volume; in other words, the CVD process occurs in a “digital type mode”. This variant of the CVD process is known as atomic layer deposition (ALD), whose main characteristic is to promote self-limited surface reactions, which allow us to obtain conformal coatings with uniformity superior to CVD techniques.

In the ALD technology, there are several modifications of the reactor structure in order to enable its use in low-temperature processes, among which the use of plasma (plasma-enhanced atomic layer deposition, PEALD) stands out [21]. The application determines the choice of process/reactor based on the requirements for substrate material and coating materials, surface morphology, film thickness and uniformity, availability of precursors, and cost [22].

In this context, the CVD-based deposition of SiC films is undoubtedly the fundamental technology that has allowed the advance of SiC-based MEMS/NEMS devices in recent decades [23]. In addition, the growth of high-quality SiC-doped films has enabled the progress of high-performance SiC electronics [23]. Multiple CVD-based deposition methods have been established over the past four decades, including APCVD, LPCVD, and PECVD. Each deposition method has its own set of process parameters and characteristics. SiC thin-film deposition has been most recently reviewed in detail by Wijesundara and Azevedo [7]. Although other review articles and book chapters also cover SiC film deposition technology [24,25,26,27], there are still few reports focused on presenting the progress of CVD techniques for SiC MEMS/NEMS applications. In the next topics, we will present in detail the conventional CVD techniques applied to the synthesis of SiC films, with a special focus on PECVD and ALD-based techniques.

### 2.1. Atmospheric Pressure Chemical Vapor Deposition

APCVD was a dominant technique for the growth of SiC films during the 1980s and early 1990s, mainly due to the accessibility of APCVD systems [27,28]. With the improvement of LPCVD reactors, the focus was shifted, because it has better control of the film growth in terms of gas phase nucleation and impurity levels. Most of today’s industrial processes are currently based on LPCVD, but APCVD is still being applied in research labs around the world [7].

CVD of SiC films has been carried out by decomposing several organic or organometallic compounds in the most varied types of designed reactors. In the case of APCVD, methyltrichlorosilane (MTS, CH_3_SiCl_3_) is the single-source precursor most often used in SiC film synthesis, not only due to the stoichiometry of silicon (Si) and carbon (C) in its molecular structure, but also due to the fact that good quality films are obtained [29,30,31]. Another single-source precursor recently used is the hexamethyldisilane (HMDS, Si_2_C_6_H_18_) [32]. As dual-source precursors are used in APCVD, we can cite the mixture of propane (C_3_H_8_) and silane (SiH_4_) [33] or dichlorosilane (DCS, SiH_2_Cl_2_) and trichlorosilane (HCl_3_Si) [34]. For both cases, a high-purity hydrogen and argon mixture is used as a carrier gas [30].

With regard to the experimental setup, the APCVD system is relatively simple due to the incorporation of few temperature sensitive devices. Basically, it consists of a gas and/or vapor precursor flow injection system, a gas mixing chamber, and a reaction chamber that can be heated or not, as well as an exhaust system to eliminate possible non-reacted precursors and byproducts [35]. In order to avoid contamination of external air by process byproducts, a gas scrubber is often used before the exhaust.

SiC growth rates reported in this kind of CVD reactor are of up to several μm/min [33,34], with the possibility of performing the film doping process with materials of type n or p [34]. We can cite as n-type material used in APCVD SiC as the nitrogen [36] and as p-type material as the boron [30]. In the literature, it is reported that both 3C-SiC epitaxial and polycrystalline films have been deposited by the APCVD technique. It is particularly advantageous for SiC epitaxy, as temperatures in the order of or greater than 1300 °C are normally required for the growth of single SiC crystals on silicon wafer [28].

### 2.2. Low-Pressure Chemical Vapor Deposition

LPCVD is the most common CVD system applied for the growth of SiC films. Although the deposition rates of SiC films in LPCVD processes are considerably lower compared to APCVD processes (in the order of nm/min), due to the easy scaling of vacuum system and the greater uniformity of temperature distribution in substrate holder, it is possible to cover a larger area of surface coating in LPCVD systems. Due to the vacuum system applied in the LPCVD, it reaches pressures of some orders of magnitude below the atmospheric, a fact that increases vapor phase diffusivity, which in many cases causes the deposition process to be reaction-rate-limited instead of diffusion-limited, increasing uniformity and conformality of the deposited films [25]. Low pressures also reduce the rate of gas phase nucleation and resulting particulates [25]. Furthermore, a LPCVD reactor allows operating more varieties of precursors and reduces the incorporation of impurities in the as-deposited films [28]. The only differences are related to the process reactor that must withstand a high-pressure gradient and the insertion of a vacuum system at the exhaust point of the reactor [37].

Both cold wall reactors, where only the growth substrate and chuck are heated, and hot wall reactors, where the entire reactor is externally heated, have been used for CVD of SiC [25].

Through of LPCVD technique, 3C-SiC epitaxial films were grown on Si wafers [38]. In recent years, LPCVD has become a leading technique for the growth of 3C-SiC polycrystalline films on various substrates, including silicon dioxide (SiO_2_) [39] and silicon nitride (Si_3_N_4_) [40]. SiC films are deposited in LPCVD using dual-source precursors, i.e., one for Si and other for C. Several chemicals have been implemented in this technique, such as SiH_4_ or DCS as a source of Si and C_3_H_8_ or acetylene (C_2_H_2_) as a carbon source [41,42,43,44,45,46]. Single-source precursors, including MTS [47], methylsilane (CH_6_Si) [48,49], diethylsilane ((C_2_H_5_)_2_SiH_4_)) [50], diethylmethylsilane (DEMS, C_5_H_13_Si) [51], tetramethylsilane (TMS, C_4_H_12_Si) [52], hexamethyldisilane (HMDS, Si_2_C_6_H_18_) [52,53], ditertiarybutylsilane (C_8_H_18_Si) [54], silacyclobutane (C_3_H_6_Si) [55], dimethyldichlorosilane (DMDCS, (CH_3_)_2_SiCl_2_) [56], 1,3-disilacyclobutane [57], and 1,3-disilabutane (DSB) [58,59] are also frequently used in the deposition of SiC films via LPCVD.

The SiC doping process can also be performed in the LPCVD process using doping precursors, such as 1,3-disilabutane, nitrogen (N_2_), ammonia (NH_3_), etc. [60]. For example, nitrogen-controlled doping has been demonstrated by the addition of N_2_ or NH_3_ as the precursor into the feed gases. By varying the dichlorosilane and 1,3-disilabutane fractions in gas mixtures, the residual stress and strain gradient of the polycrystalline SiC films were adjusted [61].

### 2.3. Metal–Organic Chemical Vapor Deposition

MOCVD is an enhanced variant of CVD that uses metal–organic chemistry for one or more of the precursors. This CVD-based technique has enjoyed steady development since Manasevit’s initial work in 1969 [62]. It is one of the CVD methods frequently applied for the synthesis of SiC films [63], especially for the growth of thick SiC films due to its higher deposition rates (in the order of 40 nm/min) [64]. Thermodynamic effects—the dynamics of the fluids inside the reactor and the reactions in the gas phase and on the substrate surface—have a synergy in the film deposition process by MOCVD. A “cold” wall reactor is usually applied for MOCVD, with the precursors being taken to the reactor/heated substrate by means of a carrier gas. MOCVD usually occurs at high temperatures (700–1100 °C) and reduced to atmospheric pressures (10–760 Torr) [65]. Under such conditions, the rates of surface chemical reactions are much faster than that of mass transport, i.e., the CVD occurs in limited mass transport-based process [65]. The reviews of Thompson et al. [63], Fischer et al. [66], Juergensen et al. [67], Creighton and Parmeter [68], and Lee et al. [69] discuss well the main reactors and growth modes of MOCVD technique.

Some MOCVD SiC processes use single-source precursors, such as DEMS [64,70,71]. No gas carrier or bubbler was thus applied. The films grown at low temperatures (850 and 900 °C) on both substrates showed crystalline 3C-SiC in the (111) orientation [64]. Homoepitaxial SiC films were grown on 3.5° off-oriented (0001) 6H-SiC by MOCVD using bis-trimethylsilylmethane (BTMSM, C_7_H_20_Si_2_) [72].

### 2.4. Plasma-Enhanced Chemical Vapor Deposition

PECVD is a modification to the CVD system, where the plasma is used to enhance the decomposition of the reactive gas source. In this process, different kinds of ions and radicals formed from chemical reactions in the plasma (mainly electron impact ionization and dissociation) diffuse toward the substrate where chemical surface reactions are promoted leading to film growth. The lower temperatures used in PECVD are its major advantage compared to conventional CVD. The gas and surface reactions occur by thermal activation in CVD, whereas in PECVD the electron temperatures of the order of 2–5 eV is enough for dissociation. This helps the coatings that are difficult to form by CVD due to melting problems, as they can be deposited by PECVD. The main types of plasma sources used in this application are the radiofrequency (rf) discharges, pulsed discharges, and microwave discharges. For more details concerning the PECVD reactors and mechanisms, see references [8,24,73,74].

The growth of SiC has been carried out in conventional PECVD reactors with or without heated substrate holders. Moreover, the low temperatures (between 200–600 °C) during PECVD processes make it feasible to deposit SiC on a variety of materials (e.g., aluminum), which is not possible using APCVD and LPCVD processes. The low deposition temperatures also confirm its potential suitability for some thermal sensitive polymer processing [75]. As hydrogenated precursor gases are used in the SiC growth by CVD processes, significant amounts of hydrogen (free and bonded) are incorporated in SiC films deposited at a low temperature (below 600 °C) by PECVD. The as-deposited low-temperature PECVD SiC films are generally amorphous (a-SiC), and thus post-deposition annealing is required for crystallization. Otherwise, there is the problem of contamination for the grown films. However, this certain method often results in uncontrolled contamination, for example, by oxygen from water vapors easily absorbed on PECVD chamber walls, present in residues of atmosphere after sample loading, and especially in precursor gases due to insufficient cleanness. Particularly, such effect was mentioned in [76] where nonstoichiometric a-SiC films were studied, and even high-purity precursors did not allow to obtain films without oxygen. Such (and other) contamination can reach several at. % and, evidently, substantially influences the films properties. It is important to note that this phenomenology is not exclusive to CVD processes but occurs in physical vapor deposition (PVD) processes as well, when operated at low temperatures, thus forming SiC films containing oxygen and/or nitrogen, that are inserted in growing film unintentionally [77].

On the other hand, crystalline SiC films were deposited by PECVD at temperatures of 650–1000 °C [78]. In recent work, Zhuang et al. reported the growth of three different kinds of 3C-SiC films, namely nanocrystalline, microcrystalline, and epitaxial (001) 3C-SiC films using microwave plasma chemical vapor deposition (MWCVD) techniques [79].

In PECVD of SiC films, methane (CH_4_) and silane (SiH_4_) are commonly used as carbon and silicon precursors, respectively [80]. In addition, the synthesis of PECVD SiC films using a single precursor, such as DSB, methylsilane, MTS, or HMDS, have also been reported [26,81,82,83]. Due to the importance of SiC produced by PECVD, a recent theoretical study discussed the reaction of various silicon and carbon precursors with bare 3C-SiC (011) surfaces (where silicon and carbon atoms are exposed) and H-terminated 3C-SiC (011) surfaces and how silane plasma fragments react with H-Si and H-C bonds of the H-terminated 3C-SiC (011) surfaces [84]. From this work, several pathways for ALD of SiC were proposed.

Doped and undoped SiC films can be synthesized by PECVD [85,86]. SiC-based thin films, such as silicon carbon nitride (SiCN), silicon oxycarbide (SiCO), silicon carbon oxynitride (SiCNO), silicon borocarbide (SiCB), silicon borocarbide nitride (SiCBN)s and silicon phosphorus carbide (SiCP), have been extensively investigated to use either as a semiconductor or as an insulator in electronic and MEMS devices. Promising applications in diodes, thin-film transistors (TFTs) and MEMS sensors have been reported [86].

Among the SiC-based films, SiCO and SiCN are extensively studied and present improved properties in comparison with pure SiC material, such as tuned bandgap and Young modulus for MEMS and NEMS applications. Silicon oxycarbide films have been grown predominantly through low-temperature PECVD using an array of silane-based precursors [87]. In the growth of SiCO films by low-temperature PECVD, an array of silane-based precursors have been investigated. However, it has been observed the incorporation of source precursor fragments and decomposition byproducts in the deposited films, which resulted in enhanced stress levels and increased defect density, both of which affecting the optical performance of the materials and device structures. Recently, Lin et al. reported the growth of amorphous SiC_x_O_y_ by very high-frequency PECVD technique [88]. An extensive review on the synthesis and properties of SiC_x_O_y_ films is presented in [89]. In addition, SiCN films have been preferentially grown by PECVD methods. Different physical and chemical deposition processes of SiC_x_N_y_ films and the main results were reviewed in [90]. In the synthesis of SiC_x_N_y_ films, several precursors have been used, including single-source precursors, such as hexamethyldisilazane, bis(dimethylamino)dimethylsilane, tris(dimethylamino)silane, tris(diethylamino)silane, and multicomponent gas mixtures—SiH(CH_3_)_3_ + N_2_ + H_2_ + Ar, SiH_4_ + CH_4_ + N_2_, Si(CH_3_)_4_ + NH_3_, etc. [91].

### 2.5. Atomic Layer Deposition

Although the atomic layer deposition (ALD) technique has been developed in the 1960s and 1970s, it was only after the rise of nanotechnology that it became more investigated, especially to grow high-quality high-k gate dielectrics for applications in transistors and nanoelectronics devices [92]. Nowadays, ALD has emerged as an outstanding technique for processing materials at the atomic level [93,94].

Recently, the articles of Ovanesyan et al. [11] and Filatova et al. [10,84] reviewed and discussed the ALD of SiC films. They stated that this process is extremely challenging for carbide material deposition. In fact, the ALD process is more commonly used for the deposition of oxides, nitrides, and sulfides. The few existing thermal processes for SiC thin-film deposition reported as ALD in the literature were operated in LPCVD reactors at substrate temperatures between 750–1200 °C and by using SiH_2_Cl_2_ or Si_2_H_6_ as silicon precursor and C_2_H_2_ or C_2_H_4_ as carbon precursor [95,96,97,98,99,100,101,102,103]. Only the work of Sadayuki et al. that presented a SiC ALD process at a relatively low temperature (590–675 °C) using diethylsilane as a single-source precursor and a growth per cycle (GPC) of 0.1 nm/cycle [103]. On the other hand, the high-temperature processes reported GPC values much greater than 0.2 nm/cycle. This suggests that during the film they may not be self-limiting likely due to the influence of a CVD component.

An important consideration is that there are reports of ALD processes with temperatures greater than those used in thermal decomposition of the surface species created after the reaction of Si_2_H_6_, C_2_H_2_ and C_2_H_4_ precursors [11,104]. In particular, the desorption of H from Si surfaces begins to occur at temperatures above 350 °C and has also been reported that, above 423 °C, methyl adsorbed groups on an Si surface begin to decompose through a mechanism of elimination of H [11,104,105]. This suggests that growth is occurring through pulsed CVD, where the GPC is controlled by the precursor exposure and not by self-limiting surface reactions. Nagasawa et al. did not observe a substantial reduction in the deposition temperature using a chlorosilane precursor [96,97], whereas Sadayuki et al. proposed an alternative approach using a single-source precursor, SiCl_2_(CH_3_)_2_, that contains both Si and C atoms [103]. However, as stated by Ovanesyan et al. [11], it is not clear how a single-source process can be self-limiting, especially at the reported growth temperature.

In order to evaluate possible precursors for a low-temperature SiC ALD process, Filatova et al. used ab initio density-functional theory (DFT) to calculate the Gibbs free energy (ΔG) of reactions for a broad spectrum of Si and C precursors for the low-temperature thermal (400 °C) ALD of SiC [10,11]. Their results suggest that, in terms of thermodynamics, SiH_4_, Si_2_H_6_, or SiH_3_Cl as silicon precursors combined with C_2_H_2_, CCl_4_, or CHCl_3_ as carbon precursors are the most promising precursors for the ALD of SiC at 400 °C. Nevertheless, experimental tests showed that some precursors with the most negative ΔG do not lead to ALD of SiC at low temperature [10]. Therefore, in order to activate SiC ALD at low process temperatures required by the current MEMS and NEMS technologies, new chemical routes or silicon and carbon precursors are likely to be needed.

Regarding the industrial applications, initially the ALD was used almost all based on the growth of binary compounds, particularly metal oxide or nitride. It is estimated that more complex materials, such as doped, ternary, and quaternary compounds, will be introduced in a next step [106]. The difficulty of depositing a binary carbide, such as SiC film, using thermal ALD processes have made researchers look for alternative solutions, such as the incorporation of carbon in silicon-based films, such as SiO_2,_ to form ternary mixtures, namely SiO_x_C_y_ [107,108]. Zhou and Bent reported on the carbosiloxane thin films grown by molecular layer deposition (MLD) using 1,2-bis[(dimethylamino)-dimethylsilyl]ethane (DDSE) and ozone precursors at a process temperature of 110 °C [107]. They demonstrated that the SiO_x_C_y_ films can be grown in real ALD mode [109]. Moreover, thermal stability studies demonstrate that the films are stable to 400 °C, with little thickness loss even at 600 °C, which is comparable to carbosiloxane films deposited by other techniques. In recent work, Closser et al. reported on MLD of ultrathin films of methylene-bridged silicon oxycarbide using bis(trichlorosilyl)-methane and water as precursors at room temperature [108]. They verified that saturation for both precursors and a constant growth rate of 0.5 ± 0.1 Å/cycle indicate self-limiting MLD. Wang et al. recently investigated the thermal ALD of carbon-doped SiO_2_ [110]. Precursors with one Si atom and a different number (1, 2, or 3) of Si-CH_3_ groups, a different number of amino groups (1 or 2), and Si–H bonds were investigated. They found that for the monoamino, di-iso-propylaminomethylsilane (DIPAMS) precursor, good reactivity and high carbon doping could be obtained.

### 2.6. Plasma-Enhanced Atomic Layer Deposition

Plasmas cannot be used to lower the growth temperature of SiC ALD processes, because unlike SiO_2_ and SiN_x_ ALD, the reaction of plasmas containing precursors for either silicon or carbon-based precursors do not self-limit and both lead to continuous film growth [10,11]. This in turn necessitates that both the silicon and carbon-based precursor half-cycles are driven thermally.

On the other hand, PEALD has been used successfully to grow SiC_x_O_y_ and SiC_x_N_y_ films [111,112,113,114]. Ovanesyan et al. developed a novel process for the ALD of SiC_x_N_y_ films using a Si_2_Cl_6_ and a CH_3_NH_2_ plasma. They reported that under self-limiting growth conditions, this ALD process led to SiC_x_N_y_ films with up to nine atomic percent carbon with a conformality > 95% in 5:1 aspect ratio nanostructures [113]. In recent work, Y.-L. Hsu reported on the deposition of SiC_x_N_y_ films on the aluminum (Al) substrates using PEALD method and 1,3,5-Trimethyl-1,3,5-trivinylcyclotrisilazane (C_9_H_21_N_3_Si_3_, VSZ) single-source precursor in order to construct SiC_x_N_y_-based resistive switching memory [114]. They demonstrated that the PEALD tool with a simple single-precursor for SiC_x_N_y_ deposition shows excellent feasibility to be used as functional memory and selector devices, further giving the potential pathway for advanced back end of line (BEOL) process integration.

In sum, the numerous challenges associated with the ALD and PEALD process of pure SiC thin films, as well as the difficulties incorporation of C into SiO_2_ and SiN_x_ films, means that scientific breakthroughs are needed to enable the ALD of SiC, SiC_x_N_y_, and SiC_x_O_y_. These scientific breakthroughs could include substantially more reactive silicon precursors and carbon precursors, novel carbon-containing plasmas that do not lead to a-C film growth and incorporate carbon in the proper bonding configuration, or new processes that take advantage of plasma activation or thermal annealing steps.

## 3. CVD-Based SiC Requirements for Development of MEMS/NEMS Devices

SiC films are being established as materials for MEMS applications. Because of its wide bandgap (2.36 eV for 3C-SiC, 3.26 eV for 4H-SiC, and 3.02 eV for 6H-SiC in comparison with 1.12 eV for silicon), SiC has been used to manufacture electronic devices for operating in harsh environments. SiC devices have exhibited good performance at high temperatures up to 600 °C, while silicon devices, the most consolidated for different applications, operate satisfactorily only at maximum temperatures around 200 °C [115]. Other SiC attractive material characteristics include a high radiation and extreme chemical inertness, while still being RIE etchable, a high thermal conductivity (better than copper), high hardness and elastic modulus (typically ~450 GPa compared with ~130 GPa for Si), and a high critical electric field (in excess of 2 MV cm^−1^) [8,116]. In MEMS sensor applications, the 3C-SiC is the most investigated and used polytype due to its well-known CVD growth on Si wafers as well as its outstanding properties. Another advantage of the use of CVD 3C-SiC films is the low cost when compared to commercially available SiC wafer. Moreover, they can be in situ p- or n-doped by the different CVD methods, which makes them interesting tunable MEMS materials [8].

In this context, extensive research has been done on growth of SiC-based films at low- or high-temperature CVD processes aiming to produce high-quality films for application not only in the field of MEMS, but also for hard coatings [83], biotechnology [117], chemical sensors [116], and other electronic applications. At high-temperature CVD processes (>500 °C [8]), we can cite the APCVD, LPCVD, and MOCVD methods. Although still underused, the thermal ALD technique falls under the category of high-temperature process since, until now, the ALD SiC process has occurred above 500 °C [11]. At low-temperature processes (<500 °C), PECVD-based techniques are commonly used for crystalline, nano-crystalline, and amorphous SiC film synthesis. In addition, more recently, low-temperature PEALD of SiC-based materials has demonstrated potential for growth of SiC-based materials, such as SiCO and SiCN [111,112,113,114].

One of the most critical problems faced by CVD SiC films for MEMS device applications is the high temperature required to guarantee surface reactions and a good deposition rate. The low-temperature deposition is very important from the point of view of recent device integration. APCVD SiC films have high deposition rates, crystallinity, and stoichiometry [33,34], but uniformity control is not good, since it is a process controlled by mass transport. Both LPCVD and PECVD are performed at low pressure, a fact that allows better control of the uniformity of the SiC film. For LPCVD the higher process temperatures allow obtaining high-quality SiC films with well-controlled material structure, stoichiometry, and pinhole density [118]. However, the high process temperatures place limitations on the types of substrate and other materials that may be present in the samples. On the other hand, at lower temperatures, the deposition rate decreases and auxiliary energy sources, such as RF generators or microwave sources, must be added to the LPCVD reactor to improve the chemical reaction by providing radicals from a self-sustained plasma (PECVD) [8]. In many cases, the quality of as-deposited SiC films at lower temperatures is lower than that observed for high-temperature LPCVD films. An example is the increase in pinhole density. The pinholes in most PECVD processes are mostly caused by the particulate contaminants originated from reactions between precursors and radicals in the plasma–gas phase [119,120,121].

Table 1 lists the mechanical properties of SiC films produced by different CVD methods [9,91,122,123]. As can be seen, the elastic modulus and hardness of the films depend on the type of CVD method used for film deposition. Amorphous SiC and SiCN films have a higher hardness/elastic modulus (H/E) ratio than those observed for single crystal and polycrystalline SiC. The high H/E ratio evidences their high wear resistance indicating that these films may be useful for application in MEMS/NEMS devices.

**Figure 2:**
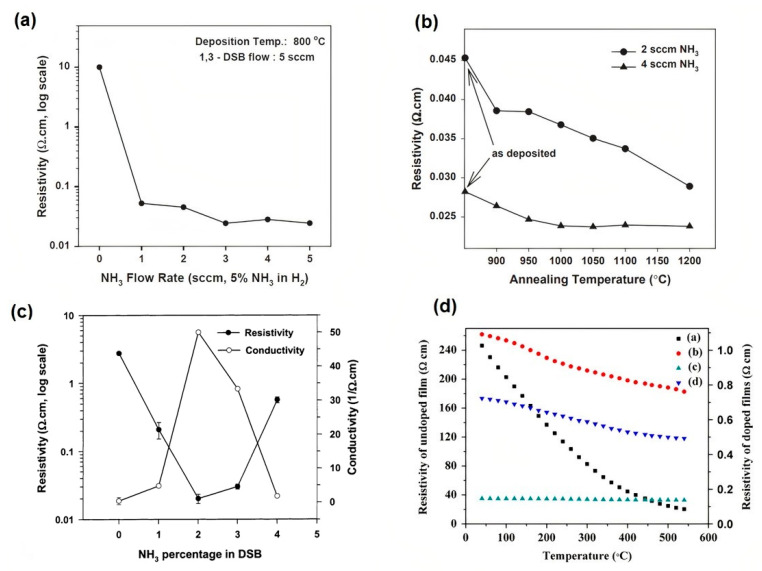
The electrical resistivity of nitrogen-doped 3C-SiC films as a function of (**a**) NH_3_ flow rate in deposition process and (**b**) annealing temperature reported by Wijesundara et al. Reproduced with permission from [124] published by Elsevier, 2003, (**c**) the resistivity and the conductivity of the 3C-SiC films as a function of NH_3_ content in the deposition process reported by Wijesundara et al. Reproduced with permission from [125] published by Elsevier, 2002, (**d**) effect of temperature on the electrical resistivity of 3C-SiC films with different nitrogen doping concentration: (**a**) 0, (**b**) 9%, (**c**) 17%, and (**d**) 30% reported by Latha et al. Reproduced with permission from [126] published by Elsevier, 2015.

In relation to electrical properties, some studies devoted to these characteristics investigated the effect of deposition conditions on the electrical resistivity. Figure 2 shows the results of three of these studies, which evaluate the influence of NH_3_ flow rate [124,125,126], deposition temperature [126], and annealing temperature [124] on the electrical resistivity of CVD SiC films. Low electrical resistivity of the order 0.02 Ω.cm was measured by Wijesundara et al. demonstrating that is possible to achieve effective doping of 3C-SiC film by optimizing NH_3_ content in the CVD process [124]. Using 17% of NH_3_ in the LPCVD process, Latha et al. obtained an electrical resistivity of 0.14 Ω·cm and a temperature coefficient of resistance (TCR) of 103 ppm/°C for 3C-SiC films grown on thermally oxidized Si substrates. These results demonstrate the potential of 3C-SiC films to be used as sensing materials in high-temperature applications.

**Figure 3:**
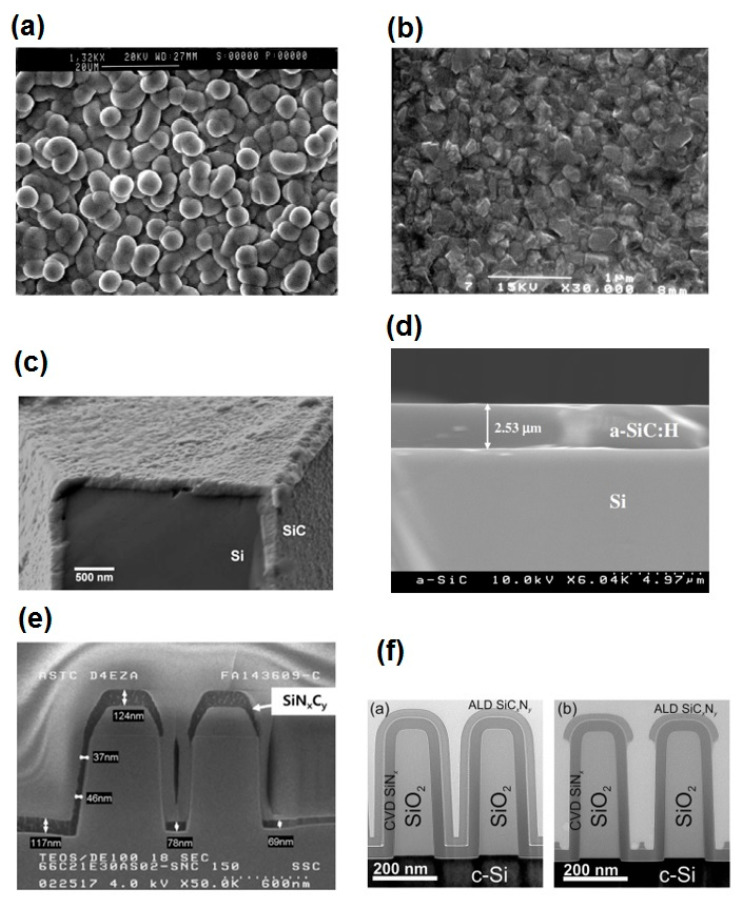
SEM micrographs: (**a**) SiC film deposited by APCVD. Reproduced with permission from [29] published by the Materials Research Society (MRS), 1999, (**b**,**c**) cross-section of SiC film deposited on a Si cantilever beam by a low-temperature CVD process. Reproduced with permission from [59] published by Elsevier, 2002, (**d**) cross-section of a-SiC:H film deposited by PECVD. Reproduced with permission from [9] published by the Materials Research Society, 2006, (**e**) cross-section of SiN_x_Cy layer deposited by PEALD. Reproduced with permission from [111] published by Elsevier, 2015, (**f**) cross-sectional TEM image of SiC_x_N_y_ film deposited by thermal atomic layer deposition (ALD). Reproduced with permission from [113] published by the American Chemical Society, 2017.

In Figure 3a–e, some micrographs of SiC-based films produced by CVD and ALD are shown, respectively. Comparing Figure 3a,b, it is possible to observe the changes in morphology of the SiC film as a function of the CVD method used, whereas the excellent conformality of the low-temperature SiC film can be observed in Figure 3c. There is no void formation at the SiC/Si interface [59]. In addition, the cross-section micrograph of PECVD a-SiC:H film (Figure 3d) shows that the film is well formed and adhered with no voids and microcracks [9]. Finally, from Figure 3e,f, it is possible to evidence the excellent step coverage of ALD and PEALD SiC_x_N_y_ films. These techniques are very promising for the advance of future NEMS devices [111,113].

## 4. Device Application of CVD SiC Films: From MEMS to NEMS

### 4.1. SiC MEMS

In parallel with the advances in the synthesis of CVD SiC-based films, microelectromechanical systems (MEMS) technology has evolved in device design, material systems, fabrication approaches and expanded its commercial applications, from automotive to consumer electronics, industry, and internet of things (IoT) [127]. MEMS devices have typical dimensions of the order of microns. They can be constituted by simple structures without moving elements or by electromechanical systems with moving elements controlled by microelectronics. Different sensing principles for MEMS devices have been demonstrated, highlighting pressure, temperature, inertial forces, magnetic fields, and chemical species [128].

**Figure 4:**
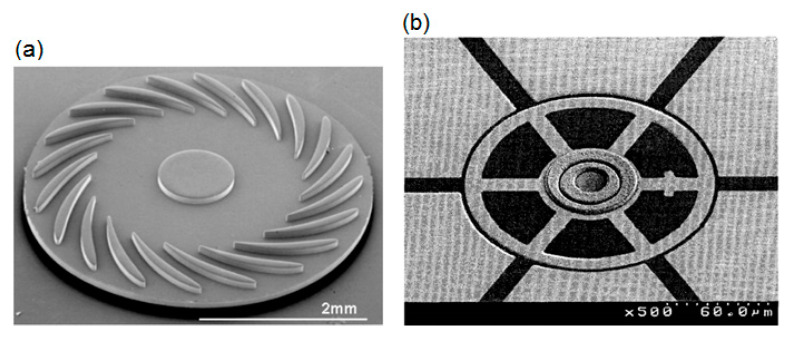
Top SEM micrograph of SiC-based MEMS: (**a**) thick shell SiC micro-turbine rotor produced in a negative mold. Reproduced with permission from [129] published by MRS, 1998, and (**b**) wobble micromotors with free bearing before dissolving mold. Reproduced with permission from [130] published by IEEE, 1999.

In the 1990s, the first articles on the use of CVD SiC films in MEMS devices were published, motivated by the need to meet harsh environment applications. One example is the growth of thick CVD SiC layers (10–100 µm) on a silicon micro-turbine rotor, which demonstrated their feasibility as a potential material candidate for the development of MEMS-based micro-gas turbine engine technology (Figure 4a) [129]. In addition, in this period, the SiC surface micromachined micromotors using micromolding and reactive ion etching (RIE) has been described, as well as the fabrication of a SiC wobble micromotor through the flange mold process (Figure 4b) [130].

Regarding the SiC MEMS sensors for high-temperature applications, mainly two types of devices started to be developed using a fabrication process based on 3C-SiC films: (i) pressure sensors of two types: capacitive and piezoresistive and (ii) cantilever resonators.

**Figure 5:**
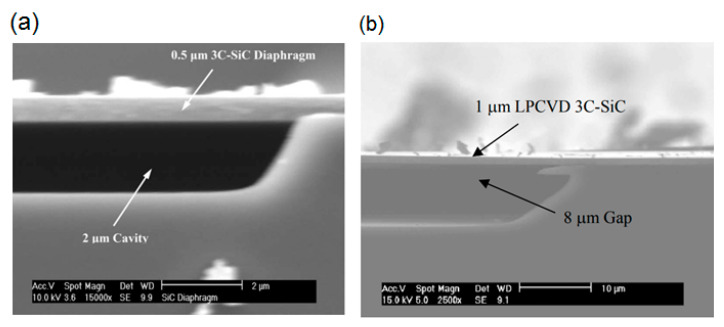
Cross-sectional SEM micrograph of SiC-based capacitive pressure sensors: (**a**) developed by Young et al. Reproduced with permission from [131] published by IEEE, 2004, and (**b**) developed by Du et al. Reproduced with permission from [132] published by IEEE, 2006.

The general structure of a SiC capacitive pressure sensor was first proposed by Young et al. and consisted of an edge-clamped circular 3C-SiC diaphragm grown by APCVD technique suspended over a sealed cavity on a silicon substrate (Figure 5a). This sensor demonstrated a good performance up to 400 °C. In addition, the authors observed that sensors with different specifications, such as linear ranges, sensitivities, and capacitance values, can be achieved by choosing the proper device geometrical parameters. However, a high residual stress was observed (>200 MPa) in the 3C-SiC film with a thickness of 0.5 µm, which restricts the design of these sensors [131]. In a subsequent paper, Du et al. reported a solution for this problem developing a capacitive pressure sensor based on low-stress heavily doped polycrystalline 3C-SiC films deposited by LPCVD (Figure 5b) [132]. They demonstrated that low-stress nitrogen-doped 3C-SiC films enables the fabrication of diaphragms with greater thicknesses. Besides of single and poly 3C-SiC films, a low-temperature SiC film deposited by PECVD has also been used to fabricate capacitive pressure sensors. Tang et al. reported a complementary metal-oxide semiconductor-compatible absolute capacitive pressure sensor for harsh environmental applications based on this material, which has good sensitivity and linearity [133].

The piezoresistive pressure sensors are, another type of MEMS sensor, commonly developed using CVD SiC films. A high-temperature pressure sensor with 3C-SiC piezoresistors sensing elements produced by LPCVD selective deposition of 3C-SiC on a patterned Si/SiO_2_ surface was developed by Eickhoff et al. [134]. The contribution of this study was to demonstrate the potential of SOI substrates as a basis for SiC thin-film sensing elements as well as demonstrated that the selective deposition process of sensing elements is a strong simplification in comparison to conventional deposition and structuring techniques. Wu et al. also developed a pressure sensor using 3C-SiC thin-film piezoresistors on a SiO_2_/Si substrate. However, the 3C-SiC film was deposited by APCVD and the sensor fabrication was carried out using wafer bonding and silicon bulk micromachining techniques [135]. Using a sensor layout similar to the proposed one by Wu et al. [135], Fraga et al. fabricated a piezoresistive pressure sensor consisting of PECVD SiC thin-film piezoresistors on SiO_2_/Si substrate [136]. This sensor exhibited performance comparable to those observed for crystalline 3C-SiC films. Furthermore, it exhibited a performance satisfactory at temperatures up to 300 °C [137]. In an article published this year, Middelburg et al. proposed a piezoresistive pressure sensor for all-SiC monolithic integration, where the piezoresistive element is an in situ, doped 3C-SiC self-sensing membrane. In this device, the polycrystalline 3C-SiC film was deposited by LPCVD and the surface micromachining was used to fabricate free-standing high topography cavities [138]. They concluded that the self-sensing concept, which enables omission of discrete piezoresistors on the membrane, has advantages, for instance it allows the tailoring of device geometry to match the performance with different applications, and the convenient processing favors the future integration with SiC CMOS, for example for impedance read-out, analog to digital conversion, or signal amplification [138].

**Figure 6:**
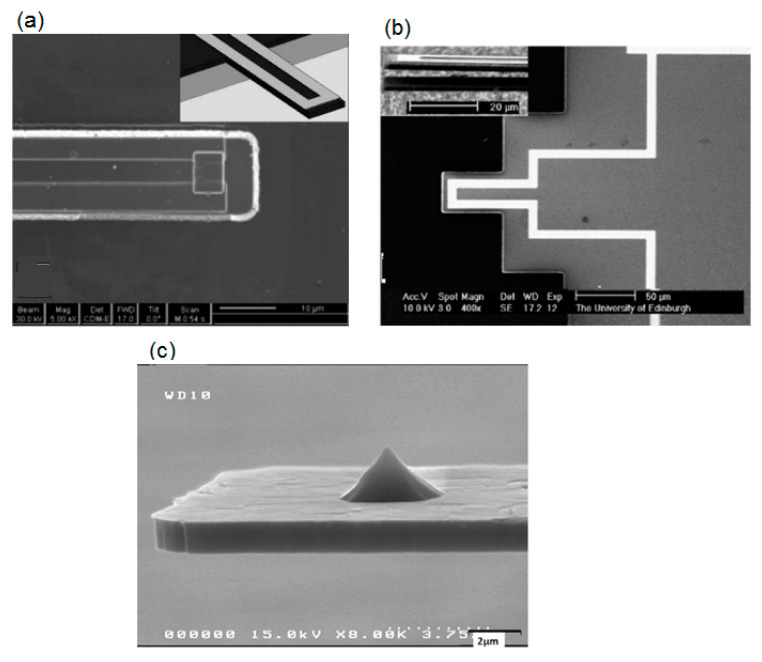
Top SEM micrograph of SiC cantilever resonators: (**a**,**b**) developed by Jiang et al. Reproduced with permission from [139] published by Elsevier and (**c**) developed by Jiao et al. Reproduced with permission from [140] published by Elsevier.

In relation to SiC cantilever resonators, Jiang et al. designed, fabricated, and tested Pt electrodes on single crystal 3C-SiC cantilevers and NiCr electrodes on poly-crystalline 3C-SiC cantilevers, Figure 6a,b, respectively. They observed that electrothermal actuation of resonance was achieved for both structures [139]. In another interesting article published in 2012, Jiao et al. proposed an approach using inductively coupled plasma etching (ICP) to fabricate monocrystalline silicon tips on 3C-SiC cantilevers for the elaboration of an entire cantilever for atomic force microscopy (Figure 6c) [140]. In a review article published in 2016, Wood et al. summarized the literature on 3C-SiC resonators concluding that they are good alternatives to Si for developing MEMS-based oscillators [141]. Recent publications on 3C-SiC MEMS resonators report the fabrication and testing of monocrystalline 3C-SiC on SOI electrostatic MEMS resonators [142] and a highly doped 3C-SiC bridge resonator [143].

PECVD SiC films have also been used for the fabrication of thermally actuated MEMS. These devices, actuated by thermal expansion induced by Joule effect consisting of matrixes of free-standing a-SiC:H and a-SiC:H/SiO_x_N_y,_ cantilevers were developed by Rehder and Carreno [144].

In addition to publications on the development of different MEMS devices based on CVD SiC films, some articles have presented and discussed new fabrication methods. For example, Phan et al. described a new method to fabricate SiC MEMS structures, such as cantilever and self-sensing membrane, which consists of applying the lithography process directly on robust free-standing SiC sub 100 nm-thick membranes making possible to replace the conventional double-sided alignment by single-sided alignment only [145].

A recent publication on SiC MEMS reports a monolithic implantable neural interface consisting of a Michigan-style 3C-SiC microelectrode array (MEA) probe formed by p-type 3C-SiC epilayer grown on a SOI wafer, which was followed by a ~2 µm-thick epilayer of heavily n-type (n^+^) 3C-SiC. In this device, a thin layer of PECVD *a*-SiC is used to insulate the surface of the probe from the external environment [146].

### 4.2. SiC NEMS

In early 2000s, nanoelectromechanical systems (NEMS), a promising technology based on the MEMS has emerged to enable the fabrication of devices with at least one dimension smaller than 100 nm [147]. The continuous miniaturization of MEMS into the NEMS has allowed the development of nanodevices and nanosensors with new capabilities or improved performance, such as increases in resonance frequency and improvements in force, mass, and displacement sensitivity [148]. In 2002, Zorman and Mehregany presented an overview of the fabrication of SiC MEMS and NEMS structures in an interesting review [149]. The focus of their review was addressing the issues of bulk micromachining and surface micromachining of SiC, which hindered its use in MEMS applications. They concluded that the material characteristics that make the fabrication of SiC MEMS devices difficult made SiC a promising candidate for the fabrication of NEMS. Another article by the same authors discusses the synthesis of SiC films for MEMS/NEMS using APCVD and LPCVD deposition systems to deposit single crystalline and polycrystalline 3C-SiC films for bulk and surface micromachined devices [150]. In a subsequent review article, Zorman and Parro discussed the development of MEMS and NEMS technologies that incorporate SiC as a key component in their mechanical structure [151].

In general, the literature about NEMS technology based on CVD SiC films shows that resonators and switches are the most investigated devices to date. In 2009, He et al. reported 3C-SiC NEMS switches capable of operation from 25 to 600 °C [152]. In subsequent publications, the same research group demonstrated promising operations toward robust long-lifetime poly-SiC NEMS switches with very small dimensions [153] and a four-terminal nanoscale electromechanical switches with a novel dual-gate design in a lateral configuration based on polycrystalline 3C-SiC nanocantilevers [154].

Recently, Feng discussed the progress and emerging frontiers of resonant NEMS [155]. Two examples of SiC NEMS resonators were presented: (i) vibrating SiC nanobeams were used to demonstrate the first microwave frequency NEMS with fundamental mode flexural resonance f_0_ > 1 GHz and (ii) SiC NEMS resonators were built with excellent stability and phase noise.

## 5. Final Remarks

The trends and perspectives of the MEMS/NEMS technology involves the integration of all components onto a single substrate and the development of WBG materials in order to meet applications in harsh environments. The inherent advantages of SiC-based materials have demonstrated their strong potential to drive progress in MEMS/NEMS technologies in the forthcoming years. First, the well-controlled synthesis of SiC thick and thin films by CVD with possibilities of tuning their excellent properties by only varying deposition parameters. Second, their compatibility with conventional MEMS fabrication technologies, which make them more competitive than other WBG materials. Recent advances in CVD and ALD techniques for synthesis of SiC films, as well as the use of these materials in MEMS/NEMS applications, have motivated the writing of this review. A good overview of the research carried out in this field was done. We presented and discussed the main CVD techniques and different MEMS/NEMS devices reported in the literature. Overall, CVD SiC films appear to fulfill the requirements of MEMS/NEMS technologies.

## Figures and Tables

**Table 1:** Mechanical properties of CVD SiC-based films deposited on (100) Si substrates measured by nanoindentation technique.

Material	CVD Method	Thickness (µm)	Elastic Modulus (GPa)	Hardness (GPa)	Hardness/Elastic Modulus (H/E) Ratio	Reference
Single Crystal 3C-SiC film	APCVD	1–2	433 ± 50	31.2 ± 3.7	0.072	[122]
Polycrystalline 3C-SiC film	APCVD	1–2	457 ± 50	33.5 ± 3.3	0.073	[122]
Polycrystalline 3C-SiC film	LPCVD	0.58–0.66	236–270	25–30	0.105–0.111	[123]
a-SiC:H film	PECVD	2.5	75	8.99	0.120	[9]
a-SiC_x_N_y_ film	PECVD	1.5	180	23	0.127	[91]
